# Purification and Characterization of a Sperm Motility Inhibiting Factor from Caprine Epididymal Plasma

**DOI:** 10.1371/journal.pone.0012039

**Published:** 2010-08-10

**Authors:** Sujoy Das, Sudipta Saha, Gopal Chandra Majumder, Sandhya Rekha Dungdung

**Affiliations:** 1 Indian Institute of Chemical Biology, Kolkata, India; 2 Centre for Rural and Cryogenic Technologies, Jadavpur University, Kolkata, India; University of Helsinki, Finland

## Abstract

Several studies have been reported on the occurrence of sperm motility inhibiting factors in the male reproductive fluids of different mammalian species, but these proteins have not been adequately purified and characterized. A novel sperm motility inhibiting factor (MIF-II) has been purified from caprine epididymal plasma (EP) by Hydroxylapatite gel adsorption chromatography, DEAE-Cellulose ion-exchange chromatography and chromatofocusing. The MIF-II has been purified to apparent homogeneity and the molecular weight estimated by Sephacryl S-300 gel filtration is 160 kDa. MIF-II is a dimeric protein, made up of two subunits each having a molecular mass of 80 kDa as shown by SDS-PAGE. The isoelectric point of MIF-II is 5.1 as determined by chromatofocusing and isoelectric focusing. It is a heat labile protein and maximal active at the pH 6.9 to 7.5. The sperm motility inhibiting protein factor at 2 µg/ml (12.5 nM) level showed maximal motility-inhibiting activity. The observation that the epididymal plasma factor lowered the intracellular cAMP level of spermatozoa in a concentration-dependent manner suggests that it may block the motility of caprine cauda spermatozoa by interfering the cAMP dependent motility function. The results revealed that the purified protein factor has the potential of sperm motility inhibition and may serve as a vaginal contraceptive. The antibody raised against the MIF-II has the potential for enhancement of forward motility of cauda-spermatozoa. This antibody may thus be useful for solving some of the problems of male infertility due to low sperm motility.

## Introduction

Reproduction is a normal process of life. Sperm maturity/motility is essential to acquire fertilizing ability of male gamete. When mammalian sperm first enter the epididymis from the testis they are neither motile nor fertile. As they travel down the epididymis, they gradually acquire full capacity for progressive motility [1]. Once maturation is complete, spermatozoa are maintained in a quiescent state in the cauda epididymis until ejaculation. Several reports are there that mammalian reproductive fluids contain some factors, which cause quiescence of sperm. The quiescence has been reported for the rat, mouse, hamster [2] and bull spermatozoa [3]. Wong & Lee [4] suggested that rat spermatozoa immobilized by hyperpolarization of the sperm membrane resulting from a Na^+^ influx parallel by H^+^ efflux. Studies on the bovine [5] reported clearly that its relatively low pH of about 5.5 acts in concert with some yet unidentified quiescence factor to maintain their immotile state. The quiescent state of rat spermatozoa in the distal epididymis may be dependent on a specific inhibitor protein, presumably acting at the sperm surface [6]. Usselman & Cone [7] demonstrated a high molecular glycoprotein, called “Immobilin” that immobilizes rat sperm mechanically by increasing the viscoelastic drag of rat cauda epididymal (CE) fluid. In our previous report we have discussed about the occurrence of motility inhibiting factor from caprine EP [8].

Poor sperm motility is one of the major causes of male infertility. The seminal plasma (SP) of many mammals investigated for the presence of motility inhibitors [9]. Rat seminal vesicle secretion possesses both motility promoting as well as inhibitory protein factors as resolved by gel filtration on Bio-gel P-150 [10]. Jeng *et al.* [11] have purified two sperm motility inhibitors (SMI-1 and SMI-2) form porcine seminal plasma. A sperm motility inhibitor from boar seminal plasma was also purified [12]. Bass *et al.* [13] found some non dialyzable factors in bovine seminal plasma that affect the viability and motility of spermatozoa. Human seminal plasma also contains a sperm motility inhibitor (SPMI) that originates from seminal vesicles as a 52 kDa precursor form and is degraded into smaller peptides by prostatic proteases shortly after ejaculation [14], [15]. Recently we have purified a 100 kDa sperm motility inhibiting factor (MIF) from goat cauda plasma membrane [16].

Though microscopic method is the most widely used subjective method for sperm motility analysis [17], here, other than this method, spectrophotometric methods were also used to estimate motility in terms of change in absorbance or optical density [18]. Another sophisticated instrument “Computer aided semen analyzer” (CASA) was utlilised that is based on microscopic video photographic method and used for estimating sperm “horizontal” velocity [19]. To determine “vertical” velocity of spermatozoa a unique computer-based spectrophotometric system was developed in our laboratory recently [20] and used for the purpose. This instrument has been named as “SPERMA”. Undertaking upward movement against gravity is much tougher as compared to horizontal movement; average vertical velocity is expected to be a much better identifying parameter for assessing quality of spermatozoa.

The current investigation has been undertaken for the first time to purify and characterize a potent sperm motility inhibiting factor (MIF-II) from caprine epididymal plasma (EP). Various assay procedures have been used to study the accurate level of the MIF-II activity on sperm forward motility as well as its velocity.

## Results

### Purification of motility inhibiting factor (MIF-II) from goat epididymal plasma

The summary of the purification of MIF-II from EP has been shown in Table 1. When hydroxylapatite gel adsorption chromatography was performed, 100% MIF-II activity was adsorbed by the gel. Activity was successfully eluted by the 0.5 M K-phosphate buffer at pH 7.0, with 70–80% recovery (Fig. S1.A). By this step, MIF-II was purified 18–20 folds. The active fraction from hydroxylapatite gel adsorption was further purified by DEAE-cellulose ion-exchange chromatography. MIF-II binds to the resin and activity was eluted with 0.2 M K-phosphate buffer, pH 7.5 (Fig. S1.B). MIF-II was further subjected to chromatofocusing, it showed single peak at the pI value of 4.8 to 5.1 approximately (Fig. S1.C). By these steps, MIF-II activity was purified about 770-fold.

**Table 1:** Purification of MIF-II from caprine epididymal plasma.

Fractions	Total Activity	Total Protein (mg)	Specific Activity	Recovery (%)	Fold Purification
**EP**	2786	279	10	100	1
**Hydroxylapatite gel adsorption**	2070	10.5	197	74.3	19
**DEAE Ion-exchange Chromatography**	1420	1.22	1163	50.96	116
**Chromatofocusing**	1540	0.2	7700	55.27	770

### Physical properties of MIF-II

**Figure 1:**
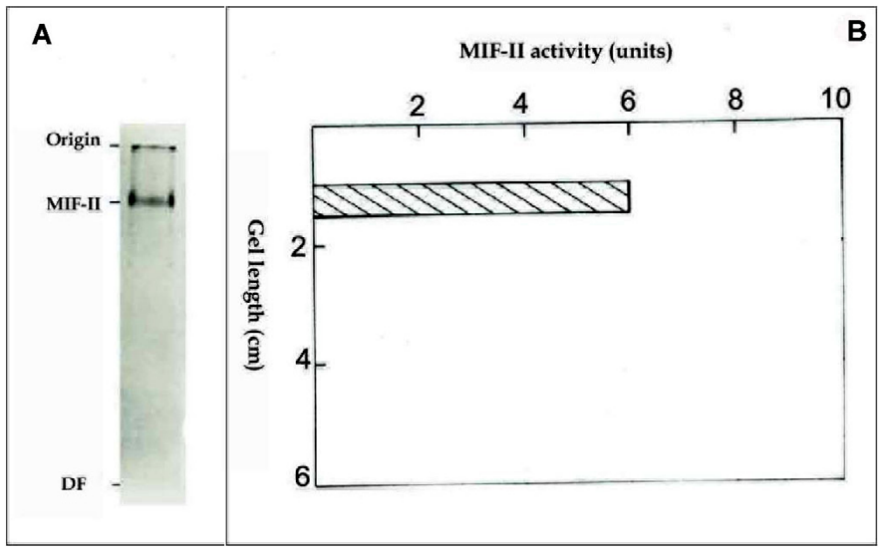
Non-denaturing polyacrylamide gel electrophoresis of the purified MIF-II on 5% gel. Staining of gel and MIF-II activity elution from gel were carried out as described in “Materials & Methods” section. A) 15 µg of purified MIF-II. B) MIF-II activity measured from the gel slices.

**Figure 2:**
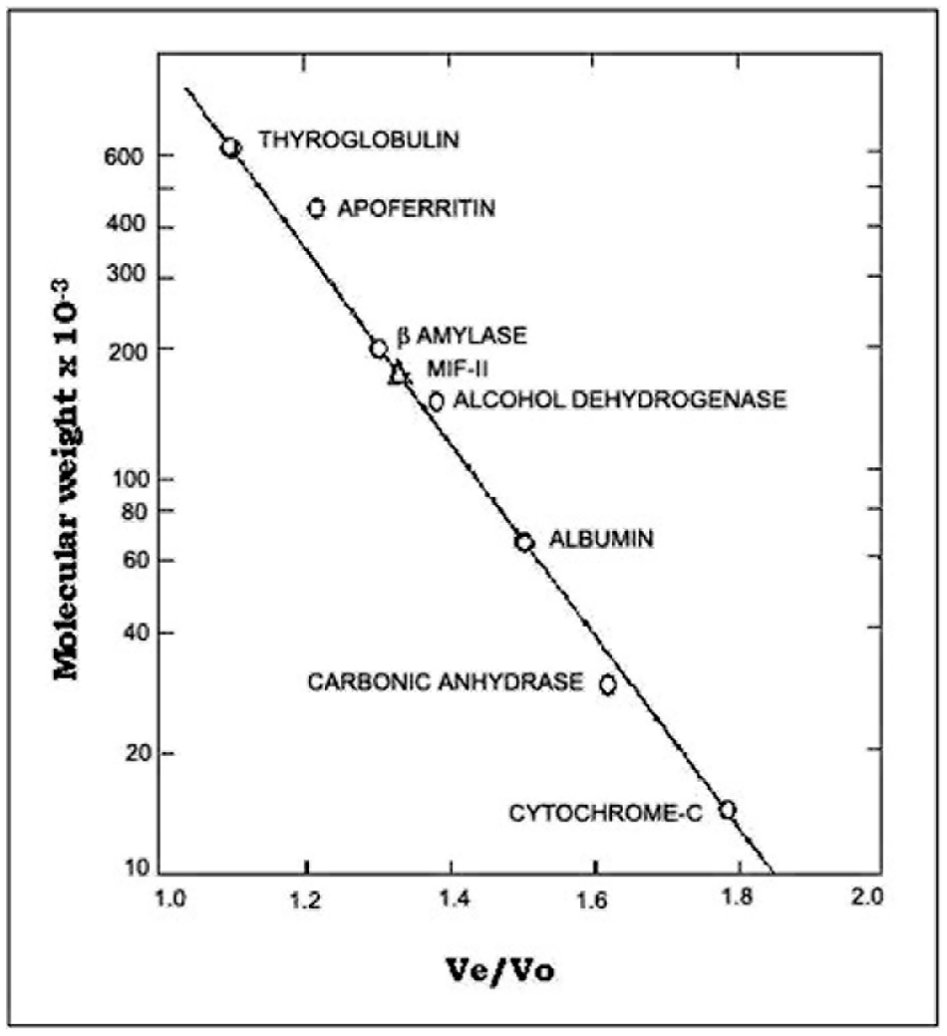
Determination of molecular weight of MIF-II by Sephacryl S-300 gel filtration. The molecular weight of MIF-II was estimated using a column of Sephacryl S-300 (1×60 cm) by modification of the method of Wollny *et al.* (1981). The Mol. Wt. Markers used as standard were thyroglobulin (669 kDa), apoferritin (443 kDa), β-amylase (200 kDa), alcohol dehydrogenase (150 kDa), BSA (66 kDa), carbonic anhydrase (29 kDa) and cytochrome C (12.4 kDa).

Purity of MIF-II was checked by native polyacrylamide gel electrophoresis. 15 µg of the purified MIF-II showed a single protein band indicating apparent homogeneity of the factor (Fig. 1A). One part of the gel was stained with silver nitrate and from another portion MIF-II activity was eluted in RPS medium. MIF-II activity co-migrated with the protein band (Fig. 1B). Molecular weight of the purified MIF-II as estimated by Sephacryl S-300 gel filtration was approx. 160 kDa (Fig. 2). Only one peak was found when MIF-II activity was subjected to gel filtration on Sephacryl S-300.

**Figure 3:**
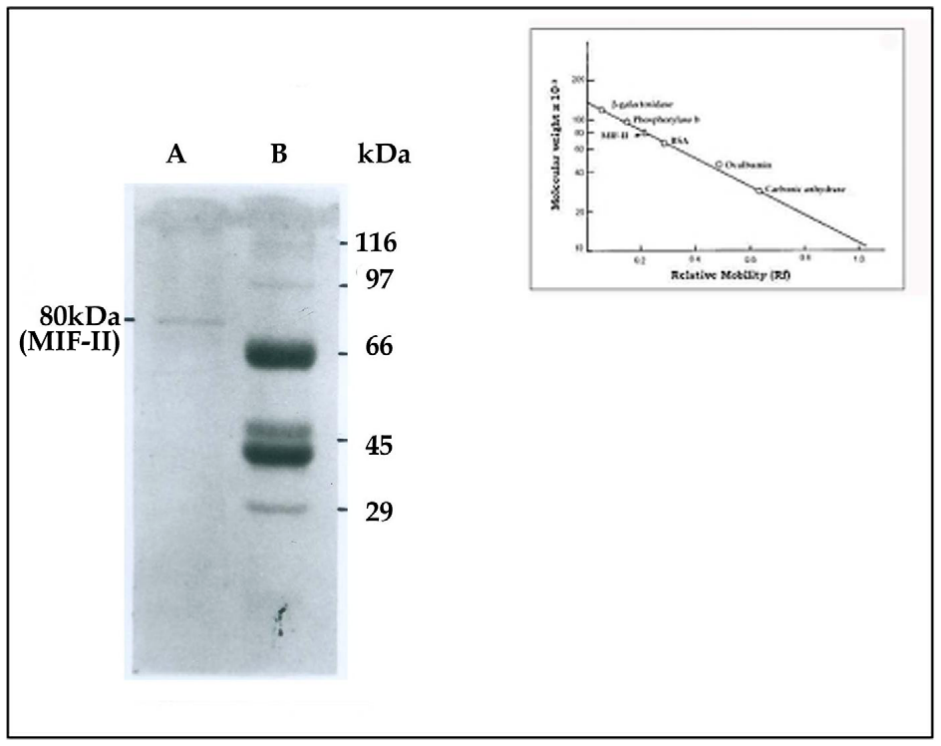
SDS-PAGE of MIF-II using 10% polyacrylamide gel. Markers used as standard were β-galactosidase (116 kDa), phosphorylase b (97 kDa), bovine serum albumin (66 kDa), ovalbumin (45 kDa) and carbonic anhydrase (29 kDa). Purified MIF-II (20 µg) was loaded.

**Figure 4:**
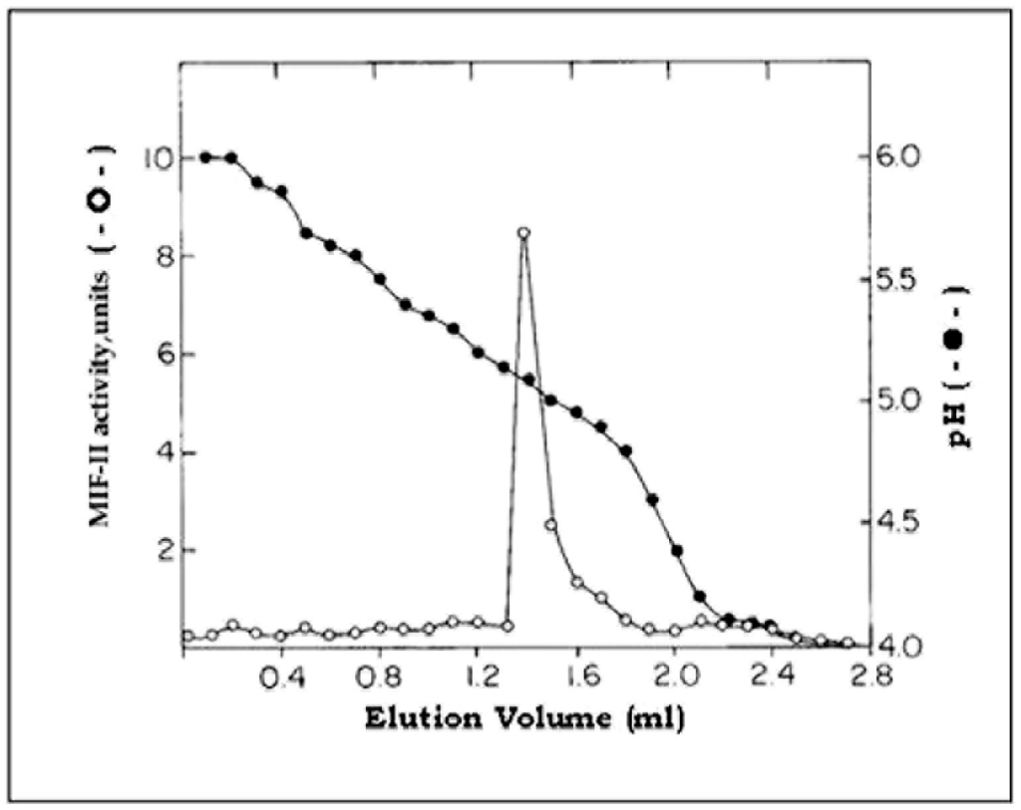
Isoelectric focusing of purified MIF-II using sucrose density gradients technique. Purified MIF-II was subjected to sucrose density gradient tube gel and focused according to the procedure described in “Materials & Methods” section.

To determine the subunit composition of MIF-II, the purified MIF-II (20 µg) was subjected to SDS-PAGE. Single protein band of 80 kDa molecular weight was observed in the gel electrophoretogram (Fig. 3). Apparent stoichiometric analysis indicated that the protein was in dimeric form. The total value (80 kDa ×2 = 160 kDa) was also consistent with the molecular weight obtained by the gel filtration chromatographic technique. The result indicates that MIF-II is a homodimer of two 80 kDa peptides. The isoelectric point of MIF-II is around 5.1 as obtained in chromatofocusing (Fig. S1.C). Using isoelectric focusing the pI point was further confirmed as 5.1 (Fig. 4).

### Biochemical Properties of MIF-II

**Figure 5:**
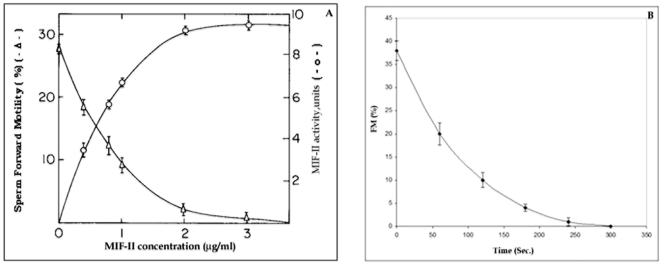
Dose course and time course of MIF-II. A) Dose course of action of purified MIF-II under the standard assay conditions. B) Time course of the action of MIF-II under the standard assay conditions. Amount of MIF-II used was 2 µg/ml. The data shown are mean ± SEM of three experiments.

**Figure 6:**
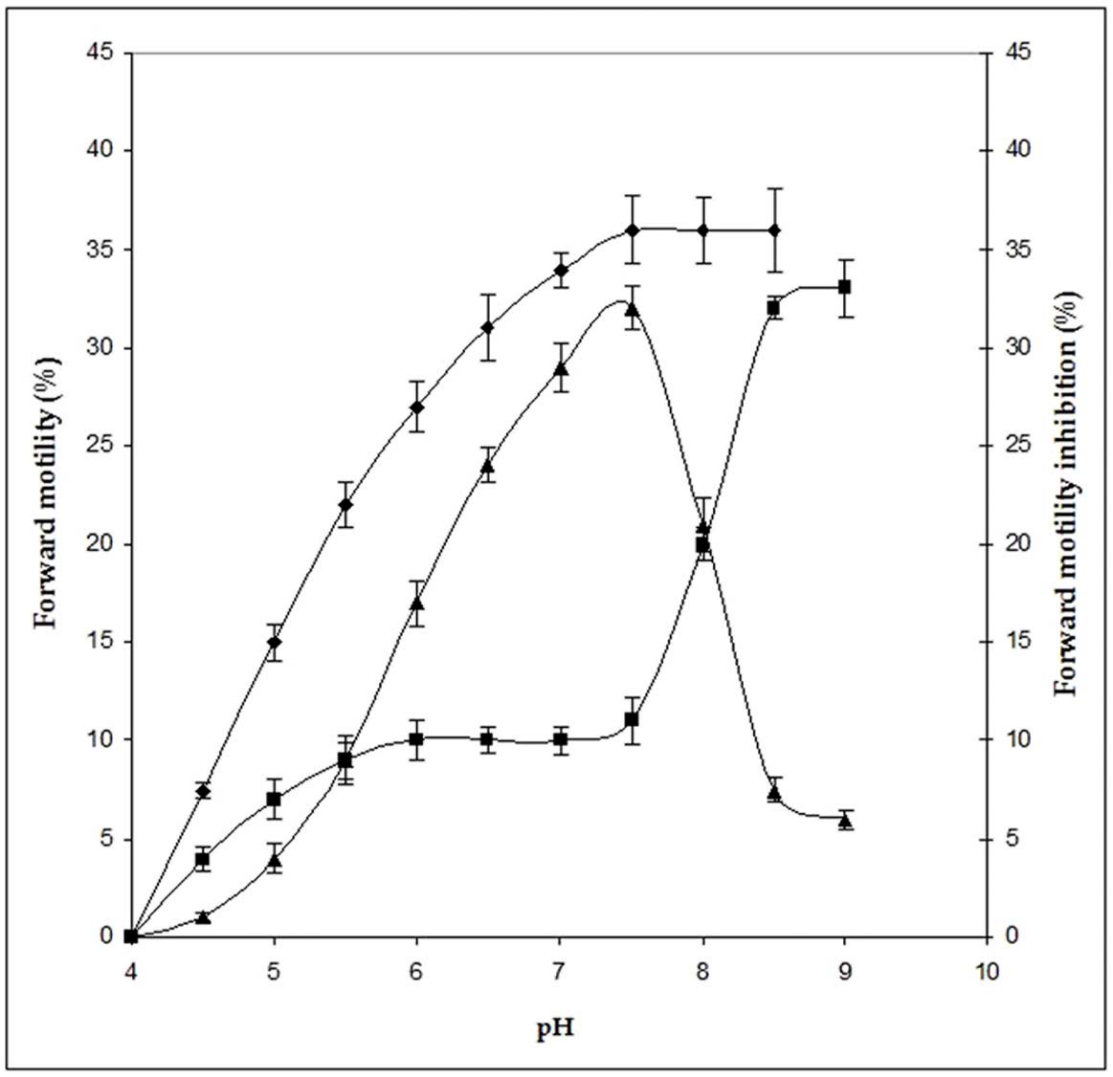
Effect of pH on the activity of MIF-II. Standard assay conditions were used except for the variation in pH of the RPS medium. Amount of MIF-II used was 2 µg/ml each. The data shown are mean ± SEM of three experiments. (♦): Control; (▪): MIF-II treated (% FM); (▴): MIF-II treated (% FM inhibition).

The motility inhibiting activity of purified MIF-II increased linearly up to 6.5 units at the concentration of 1 µg/ml (6.25 nM). The inhibitory effects of the factor showed maximal activity (approx. 92%) at 2 µg/ml (12.5 nM) concentration of MIF-II (Fig. 5.A). Fig. 5.B shows the time course of the MIF-II activity. The factor shows linear decrease of sperm forward motility and it completes within 5 min. MIF-II (10 µg/ml) when heated at 60°C, 80°C and 100°C for 2 min, cooled at room temperature and then assayed for motility inhibiting activity under the standard assay conditions, showed complete loss of activity at 100°C (Table 2). The data shows that MIF-II is heat labile protein. To elucidate the effects of pH upon the MIF-II activity, the motility of cauda epididymal sperm was studied in presence and absence of MIF-II in different pH. The motility was suppressed at the lower pH 4.0 to 4.5 in control as well as in treated sample. Observed optimum pH of MIF-II activity was 6.9 to 7.5. In alkaline pH 7.5 onwards, the MIF-II activity was lost (Fig. 6).

**Table 2:** Effect of heat treatment on motility inhibiting activity of purified MIF-II.

Heat treatment (°C)	MIF-II activity (units) (Mean ± SEM)
Nil	7.3±0.28
60	2.8±0.41
80	1.6±0.32
100	0

### Sperm motility assay by various motility analyzers

#### Analysis by CASA

**Figure 7:**
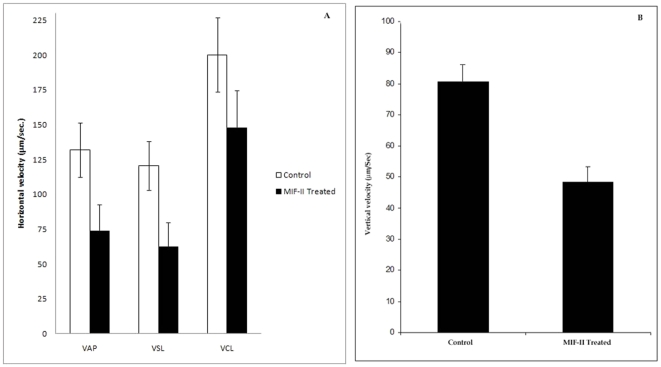
Assessment of MIF-II activity using CASA & SPERMA. A) Effect of MIF-II (1 µg/ml) on different CASA parameters. Data represents mean of three experiments (n = 3) ± SEM. Units: VAP (Average Path Velocity)  =  µm/sec, VSL (Straight Line Velocity)  =  µm/sec, VCL (Curvilinear Velocity)  =  µm/sec. When treated with 2 µg/ml of MIF-II velocity becomes undetectable. B) Effect of MIF-II (1 µg/ml) on vertical velocity of goat cauda sperm measured by SPERMA. The values indicate the mean ± SEM of three experiments. At optimum concentration (2 µg/ml) no vertical velocity was found.

Application of 2 µg/ml MIF-II to the sperm preparation caused total immobilization of all the sperm cells. Thus, 1 µg/ml was taken as the dose to see the effect of MIF-II on motility variables of CASA. There was approx. 45% decrease in VAP, approx. 50% decrease in VSL, approx. 25%, decrease in VCL (Fig. 7.A).

#### Analysis by SPERMA

Purified MIF-II at different concentrations was tested on vigorously motile goat spermatozoa. Higher velocity cells were taken for the inhibitor assay. By applying inhibitor (1 µg/ml; suboptimal level) the vertical velocity decreased from around 80 µm/sec to 48 µm/sec (Fig. 7.B). MIF-II inhibited the sperm motility completely at optimum level (2 µg/ml), so no vertical velocity was recorded. As vertical movement is much more difficult, therefore, vertical velocity corresponds to the actual condition of the cells.

### Effect of MIF-II on human spermatozoa

**Figure 8:**
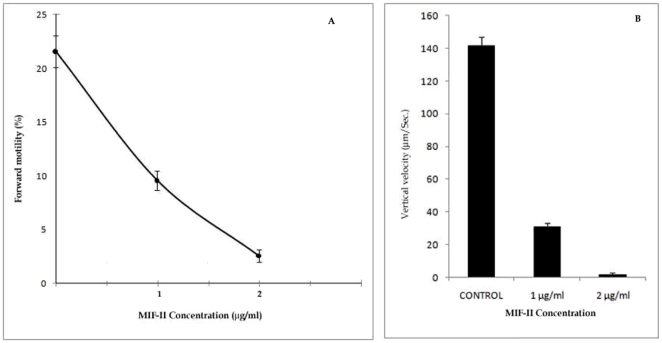
Effect of different concentration of MIF-II on human sperm. A) Effect of MIF-II on forward motility of human sperm. B) Effect of MIF-II on vertical velocity of human sperm.

MIF-II inhibits human sperm motility in dose dependent manner (Fig. 8.A). At 2 µg/ml MIF-II reduces forward motility by around 95%. When analyzed by SPERMA, it showed almost complete reduction in vertical velocity of spermatozoa at optimum concentration (Fig. 8.B).

### Intracellular cAMP concentration

**Figure 9:**
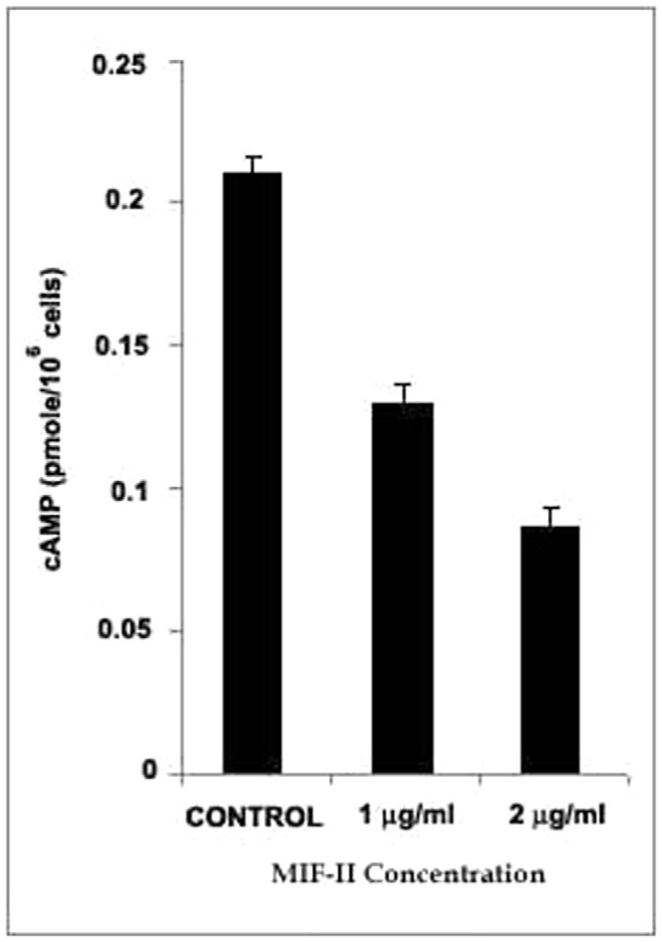
Effect of MIF-II on intracellular level of cAMP. cAMP was measured according to the procedure described in “Materials & Methods” section.

MIF-II reduces intracellular cAMP level in dose dependent manner. When cAMP_i_ was measured upon incubation with 2 µg MIF-II/10^6^ cells, it showed around 70% decrease in intracellular cAMP level (Fig. 9).

### Western blot

**Figure 10:**
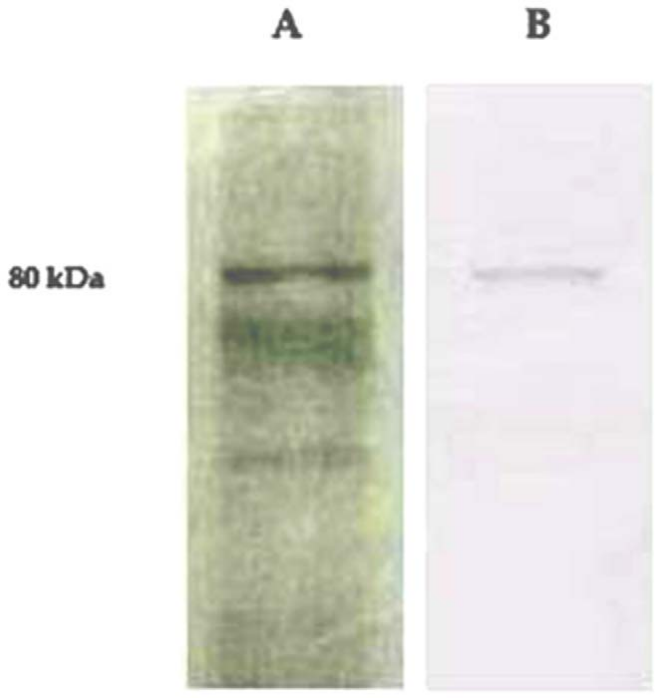
Determination of antibody specificity by Western blotting. **A**) Gel pattern of epididymal plasma (30 µg) after DEAE-Cellulose Chromatography. **B**) Western blotting of epididymal plasma after DEAE—Cellulose Chromatography.

As shown in Fig. 10.B only one stained band was obtained on nitrocellulose membrane upon Western blotting of the epididymal plasma after DEAE cellulose chromatography. The stained band corresponds to the position of the MIF-II thereby demonstrating the antibody has high immunological specificity only for MIF-II.

### Effect of MIF-II antibody on goat cauda sperm forward motility

**Figure 11:**
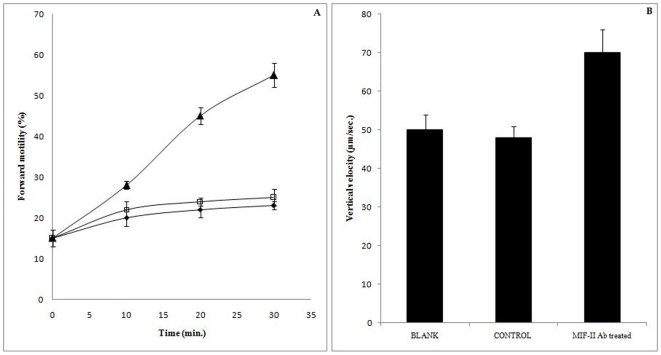
Effect of MIF-II antibody (1∶5000) on goat cauda sperm forward motility. A) Microscopic analysis: Blank (untreated) (♦), Control with pre immune sera (□), MIF-II Ab (**▴**). B) Analysis by SPERMA.

MIF-II antibody at dilution 1∶5000, promotes sperm motility by 75% as compared to the control pre-immune serum treated sperm within 30 min. of incubation (Fig. 11.A). Low motile sperm populations were taken for microscopic assay to determine the motility promoting activity of MIF-II antibody. When analyzed by SPERMA, it showed 40% increase in vertical velocity of spermatozoa as compared to the control serum (Fig. 11.B). The control serum from non-immunized rabbit did not show any effect on sperm motility. These results indicate the presence of MIF-II on sperm surface.

## Discussion

Male reproductive fluids contain a variety of biochemical components that are essential for sperm functions. Male infertility accounts for the failure of conception in approximately 30% of infertile couples [21]. One of the major causes of male infertility is due to low/no sperm motility (asthenospermia) [22]. Previous studies suggested that the seminal plasma of different species bull, boar, porcine, buffalo and human contain some sperm motility inhibitory factors [9], [11], [12], [13], [23]. Sperm motility inhibitors (SPMI) have been purified from human [14] and boar [12] seminal plasma. Occurrence of motility inhibiting protein factor has been reported in epididymal plasma of bovine [5], rat [6], [7] and caprine [8]. But there are no reports on the purification and characterization of these factors from epididymal plasma [24]. However, validity of the earliest reports on the occurrence of the motility inhibitor in different reproductive fluids remains to be proved in view of the observations that washed spermatozoa stick to the glass surface of hemocytometer which is widely used for sperm motility assays and this phenomenon of “cell- sticking” may give rise to artifact in motility estimations [25]. In the present study, sperm motility assays have been carried out in presence of goat boiled EP that contains adequate amount of antisticking factor that ruled out the possibility of “cell- sticking” artifact in motility assays. Boiled EP is devoid of motility inhibitor as MIF-II is a heat labile protein (Table 2) on other hand; antisticking factor present in EP is a heat stable protein [25]. The present investigation reports for the first time purification of sperm motility inhibiting protein (MIF-II) from caprine epididymal plasma and some of its physical and biochemical characterizations. The effectiveness and accuracy was judged by various assay procedure to prove authenticity of MIF-II.

The MIF-II was purified from caprine EP to apparent homogeneity by hydroxylapatite gel adsorption chromatography, DEAE-cellulose ion-exchange chromatography and chromatofocusing. By using these steps, MIF-II was purified to about 770 fold with 55% recovery (Table 1). The native molecular mass of the purified MIF-II is approx. 160 kDa as estimated by Sephacryl S-300 gel filtration (Fig. 2). MIF-II when subjected to denaturing SDS polyacrylamide gel electrophoresis, resolves a single protein band of 80 kDa (Fig. 3), thereby showing that the native MIF-II is a homodimer possessing two subunits each having a molecular mass of 80 kDa. The MIF-II is a heat labile (Table 2) and acidic protein as indicated by its isoelectric point around 5.1 as obtained by chromatofocusing (Fig. S1.C) and isoelectric focusing (Fig. 4). The novel motility inhibitory protein from EP is clearly different from that of seminal vesicle secretion. SP motility inhibitors (SPMI) of boar and human have pI of 8.7 and 9.0, respectively [12], [14] showing that they are basic proteins in contrast to the EP factor, which is an acidic protein. Further the EP motility inhibitor has markedly higher molecular size (160 kDa) as compared to that of seminal plasma inhibitor of boar (50 kDa) and human (18 kDa). Unlike the trimeric SP factor of boar, caprine EP factor is a dimeric protein. Biochemical basis of this marked variation of the molecular mass of motility inhibiting factors is not clear. It may be due to species variation or due to proteolytic breakdown of native protein. Previously we had purified a 100 kDa motility inhibitory factor (MIF) from caprine mature sperm plasma membrane [16]. The data demonstrate that the MIF-II is different from MIF as the sources of isolation of both the factors are different. MIF was isolated from the sperm plasma membrane whereas MIF-II is isolated from epididymal plasma. MIF (from goat sperm plasma membrane) is a heat stable, D-galactose specific glycoprotein whereas MIF-II (from EP) is a heat labile protein factor [Table 2]. The molecular mass of plasma membrane MIF is 100 kDa, which is markedly lower than that of epididymal plasma MIF-II (160 kDa). Unlike the monomeric MIF, the MIF-II is a dimeric protein. MIF-II is active at neutral pH (6.9–7.0) but it lost its activity at alkaline pH (Fig. 6) whereas the activity of sperm plasma membrane MIF is not pH dependent [16]. MIF-II not only inhibits goat cauda sperm forward motility, but also vertical velocity which is far better index of sperm quality (Fig. 7). MIF-II also inhibits human sperm forward motility as well as vertical velocity almost completely at a concentration of 2 µg/ml (Fig. 8). It implicates that though its source is caprine but its activity is not species specific. MIF-II is highly immunogenic and its antibody raised in rabbit has high immunological specificity as indicated by western blotting pattern (Fig. 10). Interestingly, antibody of MIF-II significantly increases not only sperm forward motility, but also sperm vertical velocity (Fig. 11). It is possible that the observed motility-inhibiting activity of MIF-II may be due to non-specific action of the protein. Studies were therefore carried out to estimate the forward motility-inhibiting activity of other available proteins such as BSA (66 kDa), caprine IgG (150 kDa). BSA at a concentration of 2 µg protein/ml and caprine IgG (1∶500 dilution) had no detectable effect on sperm motility (Fig. S2). However, MIF-II at a concentration as low as 2 µg protein/ml showed maximal activity when it inhibits forward motility almost completely. The results support the notion that the inhibitory action is specific for MIF-II.

Since decades, cyclic AMP (cAMP) has been reported to play an important role in both the initiation and maintenance of sperm motility [26]. Several investigators using multiple mammalian species have demonstrated that optimal *in vitro* initiation of forward motility in the immature caput-sperm requires four exogenous parameters: theophylline, epididymal plasma, bicarbonate, and alkaline pH [27]. Bicarbonate works by elevating the intrasperm level of cyclic AMP [27], [28] whereas theophylline enhances level of sperm cyclic AMP by inhibiting cyclic phosphodiesterase [27]. These findings show that elevated level of intrasperm cyclic AMP is one of the important parameters for the induction of flagellar motility. Our result demonstrates that the addition of MIF-II lowers the intrasperm cyclic AMP level up to 45% approximately (Fig. 9). It gives a justified reason for the inhibition of sperm motility due to lowering of intrasperm cyclic AMP level caused by MIF-II. Mechanism of action of MIF-II is still not clear. Increased cAMP is generally associated with increased motility [27], [28]; therefore the loss of sperm progressive motility after MIF-II treatment must have been the result of a downstream disruption in the cAMP pathway. MIF-II might be lowering the intrasperm cyclic AMP level by increasing the level of cyclic phosphodiesterase that reduces the sperm phosphorylation mechanism or reduces the level of adenylate cyclase.

There are several reports on the co-occurrence of motility initiators/promoters as well as motility inhibitors in male reproductive fluids of multiple species. These factors may work according to their level and concentration in the system. Motility initiating protein (MIP) and MIF-II both present in the caprine cauda epididymal plasma but MIP initiates the motility of caput sperm (immature) at the concentration 30 µg/ml [29] whereas MIF-II inhibits the motility of cauda sperm at the concentration 2 µg/ml *in vitro* (Fig. 5).

Population explosion is a major problem in all developing countries. As MIF-II strongly inhibits forward motility of mature sperm and its action is very specific (Fig. S2), it has the potential to be served as a contraceptive. Another global social problem of immense dimension is human infertility [21]. One of the reasons of human infertility is due to low order of sperm motility. As MIF-II antibody enhances sperm motility as well as sperm vertical velocity significantly, it has great potentiality for rectifying some of the problems of human infertility utilizing various Assisted Reproductive Technologies [30]. MIF-II antibody may also have the potential for improving cattle breeding and preservation of endangered species.

## Materials and Methods

### Reagents

DEAE-cellulose, polyethylene glycol (Mw 20 kDa), imidazole, Sephacryl S-300, gel filtration marker (MW-GF-1000), SDS marker (MW-SDS-200), phenyl methyl sulfonyl fluoride (PMSF), pepstatin A, leupeptin, Sodium dodecyl sulphate (SDS), TEMED, ammonium persulphate (APS), orthophenylene-diamine, Tween-20, AP conjugated goat anti-rabbit IgG, HRP conjugated goat anti-rabbit IgG, Ficoll-400, complete and incomplete Freund's adjuvants, gelatine, ammonium sulphate and bovine serum albumin were obtained from Sigma Chemical Co., St. Louis, MO, USA. Poly-buffer exchanger (PBE-94) and poly-buffer 74 (PB-74) were obtained from Pharmacia Fine Chemicals, Uppsala, Sweden. NBT-BCIP was obtained from MP Biomedicals, India Pvt. Ltd. Hydroxylapatite gel was obtained from Bio-Rad Lab. Other chemicals used were of reagents grade.

### Collection of caprine epididymis

Fresh epididymides of adult goats were obtained from the local slaughterhouses. Spermatozoa were extracted from the tissue within 2 to 4 hrs after slaughter of the animals.

### Preparation of spermatozoa and epididymal plasma

Spermatozoa were obtained from goat cauda epididymides as previously described [25]. Highly motile spermatozoa were extracted at room temperature (32°C ±1) from the epididymides in a modified Ringer's solution (RPS medium: 119 mM NaCl, 5 mM KCl, 1.2 mM MgSO_4_, 10 mM glucose, 16.3 mM potassium phosphate, 50 Units/ml penicillin, pH 6.9). Numbers of spermatozoa in the sample were estimated with a hemocytometer. Freshly extracted sperm preparations contained 10–20×10^7^cells/ml. For the preparation of goat cauda-epididymal plasma (EP), freshly extracted sperm preparation was centrifuged at 800×g for 10 min. The supernatant was spun again at 12000×g to obtain cell-free EP. The concentrations of EP in the assays were expressed as its protein content.

### Preparation of sperm sample for assay in CASA

The assay conditions are as same as that of forward motility assay under phase contrast microscope. Total cell numbers were counted under a phase contrast microscope at 400× magnification in a hemocytometer. Spermatozoa (0.5×10^6^cells) were incubated with boiled EP (0.6 mg protein) in absence or presence of specified amount of MIF-II at room temperature (32±1°C) for 1 min in a total volume of 0.5 ml of Ca^2+^ free modified Ringer phosphate solution. Systems lacking MIF-II served as the blanks in all assays.

### Preparation of sperm sample for vertical velocity assay by SPERMA

Extracted sperm cell numbers were counted microscopically and the concentration was made to be around 200×10^6^cells/ml with modified Ringer's phosphate buffer solution free of Ca^2+^. 400 µl of this sperm suspension and 100 µl of 10% Ficoll-400 was mixed together so that the total assay volume became 500 µl with a Ficoll-400 concentration of 2% following a procedure standardized in our laboratory. 2% Ficoll has no adverse effect on sperm motility and it was done so that only the motile cells swims up and not the dead cells [20]. This makes the final solution for application in the cuvette.

### Analysis of sperm motility inhibiting activity by microscope

MIF-II activity of EP was estimated separately by evaluating forward motility (FM) of spermatozoa using hemocytometer as the counting chamber. To eliminate the possibility of artifact due to sperm adhesion to glass, motility assays were carried out in presence of boiled EP (1.2 mg protein/ml) that contained adequate anti-sticking activity to cause nearly 100% inhibition of sperm adhesion to glass [25]. Spermatozoa (1×10^6^ cells) were incubated with boiled EP (0.6 mg protein) in the absence or presence of specified amounts of test samples (goat EP) at room temperature (32°C ±1) for 5 min in a total volume of 0.5 ml of RPS medium. A portion of the cell suspension was then placed in the hemocytometer and the forward motile (FM) sperm and total number of sperm was counted under phase contrast microscope at 400× magnification. The percentage of FM sperm was then calculated. A unit of activity of the MIF-II was defined as the amount of the factor, which inhibited FM in 10% of the cells under the standard assay conditions. The calculated percentages of FM cells are given as the mean ± SEM of at least three experiments.

### Analysis of sperm motility inhibiting activity by CASA

After preparation of sperm sample, analysis was performed using the CASA system (Version: 10, HTM-IVOS CASA System, Hamilton Thorne Research, Inc., Beverly, MA, USA.) for assessing the activity of MIF-II. Briefly, a 5 µl aliquot of prepared sperm sample was placed on a Mackler chamber. At least 200 spermatozoa were counted with CASA to evaluate the sperm motility variables including VAP (Average Path Velocity), VSL (Straight Line Velocity) and VCL (Curvilinear Velocity). The CASA settings were followed according to the manufacturer's instruction.

### Assay procedure for determination of sperm vertical velocity by SPERMA

A unique computer-based spectrophotometric system (SPERMA) was developed in our laboratory to determine the average “vertical” velocity of motile cells [20]. At the beginning, the initial conditions of the spectrophotometer were adjusted. The wavelength of the spectrophotometer was set at 545 nm (produces highest peak for sperm sample). The total scan time was given within a range of about 3 to 20 minutes to obtain a saturation curve for Absorbance Vs Time. The time interval between the start of each set of scan was set at 1 minute (60 seconds) so that every individual scan for different heights (1st, 2nd, 3rd or 4th) in subsequent cycles started exactly at an interval of 60 seconds. There was no delay in time as the movements are all well synchronized. 0.9 sec. is required to move the cuvette from one height to the adjacent one and the entire up and down movement to complete one cycle of scanning takes about 6 seconds but it does not cause any interference in the scanning intervals. The cuvette was then filled with 1.5 ml of modified RPS and placed in the cuvette holder of the spectrophotometer. This was important because the light beam in normal condition must pass through the uppermost part of the solution. The computer interfacing software was initiated at this stage when the spectrophotometer recorded the reference data and paused for adding sperm sample. 50 µl of prepared sample was layered slowly at the bottom of the cuvette with the help of a Hamilton Syringe. The experimental data, Absorbance vs. Time, was acquired at four different heights of the cuvette during each cycle of time scanning and recorded by the help of associated computer.

### Human sperm motility assay

Fresh ejaculated semen was collected from normal human volunteers. It was allowed to liquefy for 1 hr and then centrifuged at 500×g for 5 minutes to precipitate the sperm cells. Then the sperm pellete was washed twice in modified Ringer's solution free of Ca^2+^ and ultimately diluted to the required concentration.

The microscopic and spectrophotometric assay methods were similar to that of goat sperm assay already been described. Microscopic assays were done in Makler Chamber and assay volumes were modified according to the availability of sperm.

### Purification of sperm motility inhibiting factor (MIF-II)

Dialysed epididymal plasma was subjected to hydroxyl-apatite gel adsorption chromatographic column (2.5×1.5 cm) pre-equilibrated with 10 mM K-phosphate buffer, pH 7.0. After passing the sample, column was washed with 10 mM K-phosphate buffer, pH 7.0, and eluted successively with 0.1 M, 0.25 M, 0.5 M and finally with 1 M K-phosphate, pH 7.0. Active fraction was concentrated with polyethylene glycol and then dialyzed extensively against 10 mM K-phosphate buffer, pH 7.5, for the next step.

The resulting dialyzed MIF-II fraction was subjected to ion exchange chromatography column of DEAE-cellulose previously equilibrated with 10 mM potassium phosphate buffer, pH 7.5. After passage of the sample, the column was extensively washed with 10 mM K-phosphate buffer, pH 7.5 and eluted with 0.1 M, 0.2 M, 0.5 M and finally with 1 M K-phosphate, pH 7.5. The active fraction was concentrated and dialyzed against start buffer 0.025 M imidazol, pH 7.4 and subjected to chromatofocusing coloumn (0.7×10 cm or 3 ml) using PBE-94, previously equilibrated with 0.025 M imidazol, pH 7.4. Activity was eluted by polybuffer 74-HCl, pH 4 [31]. The elution was monitored by measuring pH of each fraction as well as activity of MIF-II. Active fractions were pooled and concentrated and dialyzed against RPS medium and kept at −20°C with protease inhibitors.

### Polyacrylamide gel electrophoresis (PAGE) under non-denaturing conditions

To check the homogeneity, the isolated MIF-II obtained from the final step was analysed by PAGE under non-denaturing conditions [32]. MIF-II 15 µg and 30 µg were subjected to different lane of 5% polyacrylamide gel electrophoresis without SDS. The electrophoresis was carried out towards cathode to anode using bromophenol blue as the tracking dye at 6°C with a constant current of 20 mA/gel. After completion of electrophoresis, one lane of the gel was sliced for the assay of MIF-II activity. For elution of the MIF-II activity, each gel slice (0.5 cm thickness) was crushed and dispersed in 0.2 ml of 10 mM potassium phosphate buffer, pH 7.0, overnight at 6°C and the elutes were assayed for MIF-II activity. Remaining portion of the gel was stained with silver nitrate [33] for the detection of the protein band.

### Determination of molecular weight

The native molecular weight of MIF-II was estimated by using a column of Sephacryl S-300 (1×60 cm) by modification of the method of Wollny *et al.* [34]. The gel was equilibrated with K-phosphate buffer, pH 7.0, containing 5% glycerol. Purified and concentrated MIF-II was loaded on the column. Each 1 ml fractions were collected and MIF-II activity was monitored by motility assay. The column was calibrated with known molecular weight marker, such as cytochrome C (12.4 kDa), carbonic anhydrase (29 kDa), bovine serum albumin (66 kDa), alcohol dehydrogenase (150 kDa), β-amylase (200 kDa), apoferritin (443 kDa) and thyroglobulin (669 kDa) of MW-GF-1000 kit. Calibration curve was obtained by plotting the logarithm of known molecular weights of protein versus their respective Ve/V_0_ value (Ve is the elution volume of the protein and V_0_ is the void volume of column).

### Subunit composition

To determine the subunit composition of isolated MIF-II, the factor was subjected to SDS-PAGE according to Laemmli *et al.* [32]. Markers used for determination of molecular weight of the MIF-II subunits were β-galactosidase (116 kDa), phosphorylase b (97 kDa), albumin bovine (66 kDa), ovalbumin (45 kDa) and carbonic anhydrase (29 kDa). 20 µg purified MIF-II was loaded. After completion of electrophoresis, the protein bands were detected by silver staining [33].

### Determination of Isoelectric Point

To determine isoelectric point of the purified MIF-II, isoelectric focusing in gel tube (0.6×11 cm) was carried out using a discontinuous sucrose density gradient containing 2% ampholine, pH 4.0–6.0. A 10% polyacrylamide gel base (0.6×0.5 cm) containing 2% ampholine was prepared at the bottom of the gel tubes to support the sucrose gradient. Isolated MIF-II activity was added with 20% and 15% sucrose. Sucrose gradient was formed by layering successively 1 ml each of 22%, 15% and 10% sucrose on top of the polyacrylamide gel base. Approx. 100 µg protein was loaded. Isoelectric focusing was carried out for 4 hrs at 200 V and 2 hrs at 400 V at 6°C, using 0.1N NaOH and 0.1M acetic acid as the cathode and anode buffers, respectively. After completion of run, the gel base was punctured and fractions of 2 drops each were collected. An aliquot of the fraction was assayed for MIF-II activity and the rest was diluted ten times with distilled water and pH of each fraction was measured by a microelectrode pH meter.

Isoelectric point of MIF-II was further estimated by chromatofocusing using PBE-94 [31]. After passage of the sample, the activity was eluted with elution buffer, polybuffer 74-HCl, pH 4.0. An aliquot of each fraction was assayed for activity and the rest was monitored for pH gradient using microelectrode pH meter.

### Determination of intracellular cAMP

Cyclic AMP level of spermatozoa were determined using “cAMP Enzyme Immunoassay Kit, Direct” obtained from Sigma-aldrich (cat. No.: CA200). The EIA Direct cyclic AMP kit is a competitive immunoassay for the quantitative determination of cyclic AMP in samples treated with 0.1 M HCl. Samples (i.e. spermatozoa (50×10^6^cells/ml) after treatment with HCl were acetylated as acetylation of the samples increases the sensitivity of the assay. The kit uses a polyclonal antibody to bind cAMP in a competitive manner. Samples, alkaline phosphatase conjugate, and antibody were simultaneously incubated at room temperature in a secondary antibody coated multiwell plate. The excess reagents were then washed away and substrate p-Nitrophenyl Phosphate was added. After a short incubation time the enzyme reaction was stopped and absorbance was recorded at 405 nm. The intensity of the yellow color was inversely proportional to the concentration of cAMP in either the standards or the samples. The measured optical density was used to calculate the concentration of cAMP [35].

### Raising the polyclonal antibody against the purified MIF-II

Antiserum against the purified MIF-II was raised in rabbit by four successive injections. Immunization schedule comprised of four injections at 1^st^, 7^th^, 15^th^ and 21^st^ day. First injection was given subcutaneously using 500 µg of protein in complete Freund's adjuvant. Second and third injections were comprised of 200 µg of protein each with incomplete Freund's adjuvant. Fourth injection contained 400 µg of MIF-II with incomplete Freund's adjuvant. Blood was collected from the ear vein on 27^th^ day of inoculation and serum was prepared and stored at −70°C. MIF-II Antibody titre was checked by Enzyme Linked Immunosorbant Assay (ELISA). Non-immune blood serum was collected from the same animal before starting inoculation program.

The immunoglobulin of the immune serum was precipitated twice with 50% ammonium sulfate. The final precipitate was dissolved in 0.01 M PBS, pH 7.0, and excess ammonium sulfate was removed by dialysis against the same buffer. The immunoglobulin fraction obtained after the salt fractionation was subjected to DEAE-cellulose chromatography. Unbound protein peak containing IgG was eluted with 0.01 M phosphate buffer at pH 7.0. This purified MIF-II antibody was kept in −70°C for further immunological studies.

### Enzyme Linked Immunosorbant Assay (ELISA)

50 ml of PBS (10 mM sodium phosphate pH 7.5 containing 0.9% NaCl) was added for blank and fixed amount of MIF-II was added to microtitre plate. After washing with PBS, the wells were blocked with PBS containing 3% BSA and incubated at 37°C for 1 hr. Then the 1^st^ antibody (MIF-II antibody) in PBS containing 1% BSA was added in different dilutions. Incubation and washing was done as before followed by the addition of HRP-conjugated goat anti rabbit IgG (2^nd^ antibody at a dilution of 1∶1000 in PBS containing 1% BSA). Then the plate was incubated at 37°C for 1 hr. Finally colour development was done by using 3 mM orthophenyldiamine (OPD) in 24 mM citric acid-50 mM di-sodium hydrogen phosphate containing 0.04% H_2_O_2_ (pH 5.1–5.4) in PBS. Development of colour was stopped after 30 minutes with 4(N) H_2_SO_4_ and absorbance was measured at 492 nm by ELISA reader [36].

### Western blotting

For determination of immunospecificity, MIF-II antibody was evaluated by Western blot procedure. Partially purified MIF-II (60 µg) fraction after DEAE cellulose ion exchange chromatography was run on SDS-PAGE and transferred to nitrocellulose membrane by Bio-Rad transblot apparatus. The immunoblot was carried out according to the procedure of Towbin et al. [37] Non-specific binding sites were blocked with 3% skimmed milk in TBS (10 mM Tris-HCl, pH 7.5, containing 0.9% NaCl) for 1 h at 37°C. The nitrocellulose paper was then incubated with 1^st^ antibody (MIF-II antibody); diluted at 1∶2500 in TBS containing 1% BSA, overnight at 4°C. Then the blot was washed with TBS containing 0.01% Tween-20 and after that with only TBS. After washing the blot was incubated in alkaline phosphatase-conjugated goat antirabbit IgG (2nd antibody in TBS-1% BSA at 1∶1000 dilution) for 1 hr at room temperature (32±1°C). After further washing, immunoreactive band was visualized using NBT-BCIP as a chromogenic substrate for alkaline phosphatase.

### Protein Estimation

Microgram quantities of protein were measured by rapid and sensitive method developed by Bradford, 1976 [38].

### Statistical analysis

All experiments were repeated at least five times. The data were presented as the mean ± SEM. Significance of difference between treated and control groups were analyzed by paired Student's *t*-test.

## Supporting Information

Figure S1 Purification of MIF-II by using different chromatographic methods. A) Hydroxylapatite gel adsorption. Epididymal plasma MIF-II activity was subjected to hydroxylapatite gel adsorption column. The MIF-II activity was eluted with 0.5 M K-phosphate buffer (pH 7.0). B) DEAE-cellulose ion exchange chromatography. Active MIF-II fraction eluted from first step was subjected to DEAE-cellulose ion exchange chromatography. MIF-II activity was eluted with 0.2 M K-phosphate buffer at the pH 7.5. C) Chromatofocusing of MIF-II on PBE-94 (0.7×10 cm) chromatography column as described in “Materials & Methods” section.
(2.30 MB TIF)

Figure S2 Protein specificity of MIF-II action. Among BSA (66 kDa), caprine IgG (150 kDa) and MIF-II (160 kDa), only MIF-II showed forward motility inhibition of spermatozoa.
(0.17 MB TIF)
