# Supplementary figures and images for: Purification and Characterization of a Sperm Motility Inhibiting Factor from Caprine Epididymal Plasma
Source: PLoS One. 2010 Aug 10;5(8):e12039. doi: 10.1371/journal.pone.0012039 (PMC2919373; doi:10.1371/journal.pone.0012039)

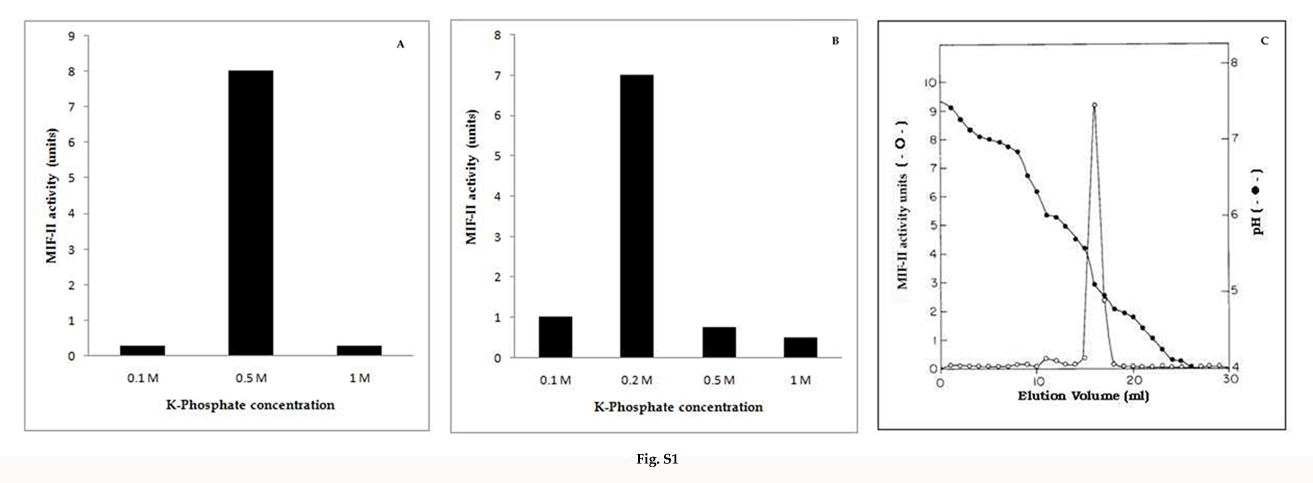

Supplement: Figure S1 — Purification of MIF-II by using different chromatographic methods. A) Hydroxylapatite gel adsorption. Epididymal plasma MIF-II activity was subjected to hydroxylapatite gel adsorption column. The MIF-II activity was eluted with 0.5 M K-phosphate buffer (pH 7.0). B) DEAE-cellulose ion exchange chromatography. Active MIF-II fraction eluted from first step was subjected to DEAE-cellulose ion exchange chromatography. MIF-II activity was eluted with 0.2 M K-phosphate buffer at the pH 7.5. C) Chromatofocusing of MIF-II on PBE-94 (0.7×10 cm) chromatography column as described in “Materials & Methods” section. (2.30 MB TIF) [file pone.0012039.s001.tif]
